# Contribution of the Community Health Volunteers in the Control of Buruli Ulcer in Bénin

**DOI:** 10.1371/journal.pntd.0003200

**Published:** 2014-10-02

**Authors:** Yves Thierry Barogui, Ghislain Emmanuel Sopoh, Roch Christian Johnson, Janine de Zeeuw, Ange Dodji Dossou, Jean Gabin Houezo, Annick Chauty, Julia Aguiar, Didier Agossadou, Patrick A. Edorh, Kingsley Asiedu, Tjip S. van der Werf, Ymkje Stienstra

**Affiliations:** 1 Centre de Dépistage et de Traitement de l'Ulcère de Buruli de Lalo, Ministère de la Santé, Cotonou, Bénin; 2 Department of Internal Medicine/Infectious Diseases, University Medical Center Groningen, University of Groningen, Groningen, The Netherlands; 3 Centre de Dépistage et de Traitement de l'Ulcère de Buruli d'Allada, Ministère de la Santé, Cotonou, Bénin; 4 Centre Interfacultaire de Formation et de Recherche en Environnement pour le Développement Durable, Université d'Abomey-Calavi, Abomey-Calavi, Bénin; 5 Centre de Dépistage et de Traitement de l'Ulcère de Buruli de Pobè, Ministère de la Santé, Cotonou, Bénin; 6 Centre de Dépistage et de Traitement de l'Ulcère de Buruli de Zangnanado, Ministère de la Santé, Cotonou, Bénin; 7 Programme National de Lutte Contre la Lèpre et l'Ulcère de Buruli, Ministère de la Santé, Cotonou, Bénin; 8 Department of Control of Neglected Tropical Diseases, World Health Organization, Geneva, Switzerland; Swiss Tropical and Public Health Institute, Switzerland

## Abstract

**Background:**

Buruli ulcer (BU) is a neglected tropical disease caused by *Mycobacterium ulcerans*. Usually BU begins as a painless nodule, plaque or edema, ultimately developing into an ulcer. The high number of patients presenting with ulcers in an advanced stage is striking. Such late presentation will complicate treatment and have long-term disabilities as a consequence. The disease is mainly endemic in West Africa. The primary strategy for control of this disease is early detection using community village volunteers.

**Methodology/Principal Findings:**

In this retrospective, observational study, information regarding Buruli ulcer patients that reported to one of the four BU centers in Bénin between January 2008 and December 2010 was collected using the WHO/BU01 forms. Information used from these forms included general characteristics of the patient, the results of diagnostic tests, the presence of functional limitations at start of treatment, lesion size, patient delay and the referral system. The role of the different referral systems on the stage of disease at presentation in the hospital was analyzed by a logistic regression analysis. About a quarter of the patients (26.5%) were referred to the hospital by the community health volunteers. In our data set, patients referred to the hospital by community health volunteers appeared to be in an earlier stage of disease than patients referred by other methods, but after adjustment by the regression analysis for the health center, this effect could no longer be seen. The *Polymerase Chain Reaction* (PCR) for IS2404 positivity rate among patients referred by the community health volunteers was not systematically lower than in patients referred by other systems.

**Conclusions/Significance:**

This study clarifies the role played by community health volunteers in Bénin, and shows that they play an important role in the control of BU.

## Author Summary

Buruli ulcer (BU) is a neglected tropical disease caused by *Mycobacterium ulcerans*. Usually, the number of patients presenting with ulcers in an advanced stage is high. This complicates treatment and increases the risk of disabilities. The disease is endemic mainly in West Africa. The primary strategy for control is early detection using community village volunteers. In Bénin, data was collected to understand the role of the different referral systems on the stage of disease at presentation in the hospital and the diagnostic precision. About a quarter of the patients were referred to the hospital by the community health volunteers. Community health volunteers referred patients more frequently in an earlier stage of disease. The PCR confirmation rate among patients referred by the community health volunteers was not systematically lower than in patients referred by other systems. We found that community health volunteers played an important role in the referral system of BU patients in Bénin. This information is relevant for health care programs attempting to control BU but may also be relevant for health care programs working on other diseases in areas with restricted resources.

## Introduction

Buruli ulcer (BU), caused by *Mycobacterium ulcerans*, is an emerging neglected tropical disease. It is the third most common mycobacterial disease after tuberculosis and leprosy in immuno-competent persons in Bénin. The disease has been reported in more than 30 countries worldwide, but the highest patient load is in West Africa [1]–[4].

Usually BU begins as a painless nodule, plaque or edema, ultimately developing into an ulcer. The high number of patients presenting with ulcers in an advanced stage is a major problem because treatment of advanced disease is complex and frequently has long-term disabilities as a consequence [5], [6]. Late presentation to health care facilities is common and is influenced by practical matters such as travel costs, visits to traditional healers and the patient's perception of illness [7], [8].

Treatment consists of antibiotic treatment using streptomycin and rifampicin for eight weeks combined with wound care till the lesion heals [4], [9]. Lesions take a long time to heal; in a clinical trial on antibiotic treatment in Ghana, the median time to healing was 18 weeks for category I lesions. The median time to healing is larger for category II and III lesions - 30 weeks [10]. Surgery may be necessary. The size of the lesion was the main factor associated with surgery [11]–[13].

The World Health Organization (WHO) recommends early case detection and early treatment of the lesions for BU control. Early case detection is important because delayed presentation for medical treatment is correlated with evolution of pre-ulcerative lesions to the ulcerative form, increased risk of osteomyelitis, more extensive surgical intervention and skin grafting [14], extended hospital admission and severe functional limitation [15]–[17]. Strategies used for early detection of BU patients are active case finding and education in the rural areas with the assistance of community health volunteers. Community health volunteers can be defined as lay individuals trained in a particular role of delivering curative or preventative care or control in their own community [18]. Community health volunteers have been shown to play an important role in controlling several endemic diseases such as onchocerciasis and dracunculiasis. To control onchocerciasis, the community health volunteers participated in ivermectin mass distribution. For dracunculiasis, they reported cases monthly [19]–[21]. In some African countries, community health volunteers contributed to the conduction of vaccine trials, and play important roles in the reduction of maternal, newborn and infant mortality as well as in the management of febrile convulsions [22]–[26].

In BU, very few studies have evaluated the role of community health volunteers. This study looks at the contribution of different actors in the current reference system in Benin, their influence on the stage of disease at presentation in the hospital and on the diagnostic confirmation rate of BU, using IS2404 PCR.

## Methods

### Management of BU control in Benin

**Figure 1:**
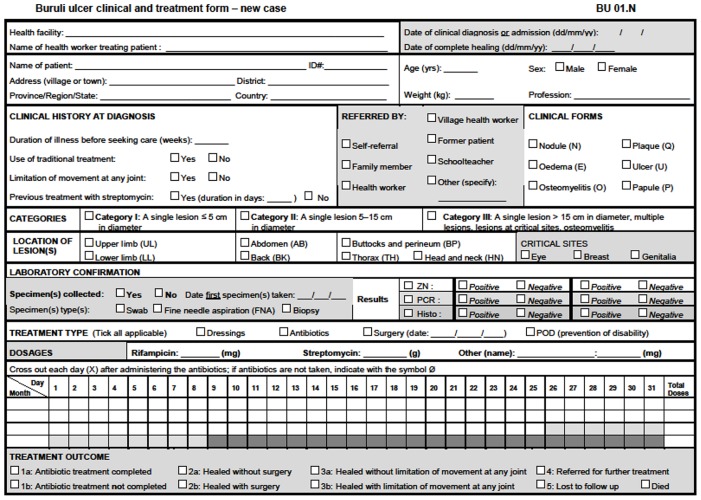
BU01 form.

In Bénin, the National Program against Leprosy and Buruli ulcer (PNLLUB) is the official body that organizes and implements BU control. Each endemic area has a Center of Detection and Treatment of BU (CDTUB) in Bénin. In total, the program has four CDTUB respectively called A, B, C and D in this study to ensure anonymity. Detection teams are composed of two community health volunteers and two teachers in each endemic village, supervised by the nurse in charge of the health area. Main roles of the detection teams are to detect patients with suspicious lesions, to refer them to the nearest CDTUB, and to follow up cured patients after care. The volunteers do not receive compensation per BU case detection. All clinically confirmed patients are registered with the BU01 form in a CDTUB. This form (figure 1) was provided by the WHO and used by all BU endemic countries to collect basic clinical data on newly registered patients for guidance of national programs since 2007 [4]. Trained nurses fill out the information on this form. Case confirmation is made by Laboratoire de Référence des Mycobactéries (LRM) in Cotonou for CDTUB A, B, D and in Anger (France) for CDTUB C. The laboratory of the Institute of Tropical Medicine in Antwerp has provided the quality control of LRM. Diagnostic tests include microscopy to detect acid-fast bacilli (AFB) and *Polymerase Chain Reaction* (PCR) for IS2404.

### Study population and procedures

In this retrospective, observational study, patient information was collected from the WHO/BU01 forms of all Buruli ulcer patients reported to one of the four BU centers in Benin between the 1^st^ of January 2008 and the 31st of December of 2010. General characteristics of the patient, the results of diagnostic tests, the presence of functional limitations at start of treatment, the category of the lesion, patient delay, earlier visits to a traditional healer and the referral system were collected from the form. Patients can be referred to health centers by a health care worker, by trained community health volunteers or teachers, or by persons not included in the detection team such as family members, former patients, or they may report themselves. These items were selected from the BU01 form because they can be useful in assessing the importance of different actors in the current reference pattern in Benin, their influence on the stage of disease at presentation in the hospital and on the diagnostic precision of BU. Community education activities and geographical and socio-economic circumstances are expected to differ between treatment centers. Therefore the treatment center together with age and sex of the patients presenting to each center were evaluated as potential confounders or effect modifiers in the relation between referral system and disease stage.

### Definitions

The presence of functional limitation at presentation was determined by visual assessment by the nurse completing the WHO form at start of treatment. Category of the lesion was registered according to the WHO classification provided by WHO [4] as follows:

- Category I. A single lesion <5 cm in diameter.
- Category II. A single lesion between 5 and 15 cm in diameter.
- Category III. A single lesion>15 cm in diameter or multiple lesions or lesion(s) at critical sites (eye, breast, genitalia), or osteomyelitis.

**Figure 2:**
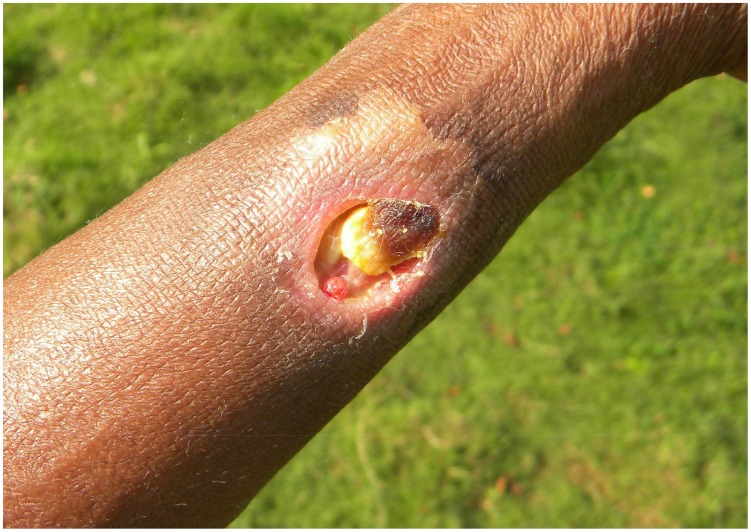
Early lesion of Buruli ulcer.

We defined an early lesion as a lesion in category 1 or 2 at admission (figure 2).

Patient delay was defined as the time between the start of the lesion (as indicated by the patient) and the date of reporting at the BU centers.

### Statistics

Statistical analysis was performed using Statistical Package for Social Science (SPSS) version 19.0. All records of BU cases treated in the treatment centers during the study period were complete for the study variables used. The records of BU patients for whom PCR results were missing, were excluded from the analysis on diagnostic precision. χ 2 tests and t tests, or Kruskal-Wallis Test as appropriate were used for the univariate analyses on patient characteristics and the referral system. The role of the different referral systems on the stage of disease at presentation in the hospital was analyzed by a logistic regression analysis (manually entered). The variables as presented in the univariate analysis were studied on confounding and effect modification.

### Ethics

The provisional national ethical review board of the Ministry of Health Benin, nr IRB00006860, approved the study protocol. All data analyzed were anonymized. For the picture include in this manuscript, written and verbal informed consent were obtained from all participants aged 15 years or older and legal representatives of participants younger than 15 years old.

## Results

In the study period, 1965 patients reported to one of the BU treating centers in Benin. The basic characteristics of the BU patients are presented in table 1.

**Table 1:** Characteristics of participants related to referral system.

	Health worker	community health volunteers	Teachers	Former patients	Family members	Self reported	Total	P-value
Age, median (IQR)	15 (7–35)	12 (7–30)	29 (9–35)	26 (12–46)	10 (6–21)	30 (15–45)	15 (8–35)	<0.001*
Sex (male %)	218 (55.1)	259 (49.7)	20 (62.5)	228 (52.7)	176 (47.8)	111 (51.6)	1012 (51.5)	0.266**
Patient delay (weeks in median,IQR)	12 (6–26)	12 (4.7–30)	13 (8.5–56)	12 (6–26)	8 (4–14)	8 (4–16)	12 (4–26)	<0.001*
Functional limitation visible at admission no (%)	83 (21.0)	67 (12.9)	7 (21.9)	57 (13.2)	132 (35.9)	61 (28.4)	407 (20.7)	<0.001**
Category 1 no (%)	48 (12.1)	138 (26.5)	5 (15.6)	17 (3.9)	94 (25.5)	50 (23.3)	352 (17.9)	<0.001**
Category 2 no (%)	142 (35.9)	217 (41.7)	14 (43.8)	139 (32.1)	131 (35.6)	82 (38.1)	725 (36.9)	<0.001**
Category 3 no (%)	206 (52.0)	166 (31.9)	13 (40.6)	277 (64.0)	143 (38.9)	83 (38.6)	888 (45.2)	<0.001**
Category 1 or 2 combined no (%)	190 (48)	355 (68.1)	19 (59.4)	156 (36.0)	225 (61.1)	132 (61.4)	1077 (54.8)	<0.001**
Visit to traditional healer before reporting to health center no (% of total by referring system)	233 (58.8)	153 (29.4)	25 (78.1)	235 (54.3)	204 (55.4)	105 (48.8)	955 (48.6)	<0.001**
Total number ( = 1965)	396 (20.2)	521 (26.5)	32 (1.6)	433 (22.0)	368 (18.7)	215 (10.9)	1965 (100.0)	

*Kruskal-Wallis Test (comparing median between authors of reference),

** Chi-square, Pearson.

Out of the 1965 individuals that presented to the health centers with BU, community health volunteers referred 521 (26.5%), former patients referred 433 (22.0%), health worker referred 396 (20.2%), and family members 368 (18.7%). Only 215 (10.9%) BU patients reported themselves. Teachers referred 32 patients (1.6%) with a median age of 29, reflecting their role in the community outside their own school with young children.

The percentage of patients that presented in an early stage of disease (category 1 or 2) statistically differed among the different referring systems (p<0.001).

Patients referred by the community health volunteers most frequently presented with an early lesion (68.1%), followed by patients referred by family members and self-reporting patients (both 61%).

Patients referred by a family member or self-reporting patients (8 weeks for both) had less delay compared to patients referred by the other actors (higher than or equal to 12 weeks). The number of patients presenting with functional limitations as well as the number of patients that visited traditional healers was lowest in patients referred by community health volunteers.

Less than a third of patients (29.4%) consulted traditional healers among patients referred by the community health volunteers.

The referral pattern also differed per center (Table 2). In CDTUB A the majority of patients (77.8%) were referred by the community health volunteers, whereas in CDTUB D the majority of patients (60.0%) were referred by a former patient. In CDTUB C and B, most patients were referred by family members, the community health volunteers or they reported themselves.

**Table 2:** Referral system and health center.

	Total number of patients	Health worker	Community health volunteers	Teachers	Former patient	Family members	Self reported	Category 1 and 2 lesions	P-value
CDTUB A no (%)	441 (22.4)	58 (13.2)	343 (77.8)	0	8 (1.8)	19 (4.3)	13 (2.9)	313 (71.0)	<0.001**
CDTUB B no (%)	425 (21.6)	62 (14.6)	95 (22.4)	10 (2.4)	40 (9.4)	141 (33.2)	77 (18.1)	283 (66.6)	<0.001**
CDTUB C no (%)	481 (24.5)	64 (13.3)	62 (12.9)	15 (3.1)	14 (2.9)	208 (43.2)	118 (24.5)	276 (57.4)	<0.001**
CDTUB D no (%)	618 (31.5)	212 (34.3)	21 (3.4)	7 (1.1)	371 (60.0)	0	7 (1.1)	205 (33.2)	<0.001**
TOTAL no (%)	1965 (100)	396 (20.2)	521 (26.5)	32 (1.6)	433 (22.0)	368 (18.7)	215 (10.9)	1077 (54.8)	<0.001**

** Chi-square, Pearson or Fisher as appropriate.

The percentages of patients reporting in an early stage of disease (category 1 or 2 lesions) differed among the centers.

The role of the different referral systems and the stage of disease at presentation in the hospital in these different groups is shown in table 3. In this model without any confounder or interaction included, referral by a community health volunteer protects against presentation of late stage of the disease (category 3), with an Odds ratios of 0.43 compared to referral by a health care worker. Table 4 shows the results of the statistical model with treatment center and age included as confounder. After correction by treatment center, the referral system does not have any statistically significant effect on the presentation of advanced stage lesions (category 3). The only statistically significant effects in the model are the high Odds ratios for presentation of advanced stage lesions in treatment centers C and D compared to treatment center A. Sex of the patient was not a confounder in the model. To obtain a workable model to control for interactions between the referral system and the treatment center, we simplified the referral system to community health volunteers versus the other referring systems. This did not reveal statistically significant interactions between the treatment center and the referral system. Early presentation can be defined as category 1 lesions only. In a post hoc analysis, this alternative definition of early disease (i.e., only category 1 lesions classify as early disease presentation) showed similar results as the model presented in table 3; after adjustment for the treatment center, there was no influence of the referral system on the disease stage patients presented to the health center.

**Table 3:** Logistic regression model assessing the referral system and severe lesion (category 3) at admission.

				95% C.I.	
	B	P-value	OR* (crude)	Lower	Upper
Health care worker			1.00		
Community health volunteers**	−0.84	<0.001	0.43	0.33	0.57
Teacher**	−0.46	0.22	0.63	0.30	1.31
Self reported**	−0.55	0.002	0.58	0.41	0.81
Family member**	−0.53	<0.001	0.59	0.44	0.78
Former patient**	0.49	0.001	1.64	1.24	2.16

* OR = odds ratio;

** Referred system as dummy variable, compared with health care workers.

**Table 4:** Logistic regression model assessing the referral system with treatment centers and age as confounders and severe lesion (category 3) as outcome.

				95% C.I.	
Variables analyzed	B	P-value	OR* (adjusted)	Lower	Upper
Health care worker			1		
Community health volunteers**	−0.14	0.421	0.87	0.62	1.22
Teacher**	−0.28	0.481	0.76	0.35	1.64
Self reported**	−0.21	0.294	0.81	0.55	1.20
Family member**	0.03	0.850	1.03	0.73	1.46
Former patient**	0.05	0.774	1.05	0.77	1.42
CDTUB A			1.0		
CDTUB B	0.10	0.551	1.11	0.79	1.55
CDTUB C	0.51	0.004	1.66	1.18	2.33
CDTUB D	1.37	<0.001	3.94	2.70	5.75
age (years)	0.01	<0.001	1.01	1.01	1.02

* OR = odds ratio; CI = confidence interval.

** Referred system as dummy variable, compared with health care workers.

PCR positivity per referral system and per health center is shown in table 5. In total, 644 of 910 patients with a sample available (70.8%) were confirmed by PCR and the percentage of confirmed patients differed among the different referral systems (p<0.001). In all, the community health volunteers referred 63.6% of the patients with a confirmed diagnosis of Buruli ulcer. Of these patients 14.2% presented with category 1 lesions and 49.4% presented with category 2 or category 3 lesions.

**Table 5:** Referral system related PCR and health center.

PCR +	Health worker	Community health volunteers	Teachers	Former patient	Family members	Self reported	Total	P-value
CDTUB A	13/20 (65.0%)	175/277 (63.2%)	N.A.	2/2 (100.0%)	5/10 (50.0%)	5/8 (100.0%)	200/314 (63.7%)	0.303*
CDTUB B	10/22 (45.5%)	5/15 (33.3%)	2/4 (50.0%)	3/17 (17.6%)	14/33 (42.4%)	5/21 (23.8%)	39/112 (34.8%)	0.338*
CDTUB C	46/56 (82.1%)	37/50 (74.0%)	10/11 (90.9%)	12/12 (100.0%)	174/189 (92.1%)	86/107 (80.4%)	365/425 (85.9%)	0.004*
CDTUB D	16/21 (76.2%)	3/4 (75.0%)	N.A.	20/33 (60.6%)	N.A.	1/1 (100%)	40/59 (67.8%)	0.566*
TOTAL	85/119 (71.4%)	220/346 (63.6%)	12/15 (80.9%)	37/64 (57.8%)	193/232 (83.2%)	97/134 (72.4%)	644/910 (70.8%)	<0.001*

* Chi-square, Pearson or Fisher as appropriate.

N.A. – not applicable.

The percentage of confirmed patients referred was different between centers: only in CDTUB C did the percentage of confirmed patients differ significantly by referral systems (p = 0.004). In the total group, patients referred by the family members are most frequently confirmed by PCR (92.1%); patients referred by former patients are less frequently confirmed by PCR (57.8%).

## Discussion

This is the first study addressing the role of community health volunteers in the control of BU in Benin. Few studies address the role of CHW in BU detection worldwide [18], [27].

The data analyzed in this study were collected in the Centers of detection and Treatment of BU (CDTUB in Bénin). Health workers were trained to fill the BU 01 form. These forms developed by the WHO have proven to be beneficial to both patient care and control by the national program [28], [29].

About a quarter of the patients were referred to the hospital by the community health volunteers. This shows that they play a major role in the control of BU. Our results confirm the impact of community health volunteers on the control of BU. The use of community health workers for BU control in countries other than Benin has been suggested [27] and also discussed in a review based on sparsely available data on their performance and training [18].

**Figure 3:**
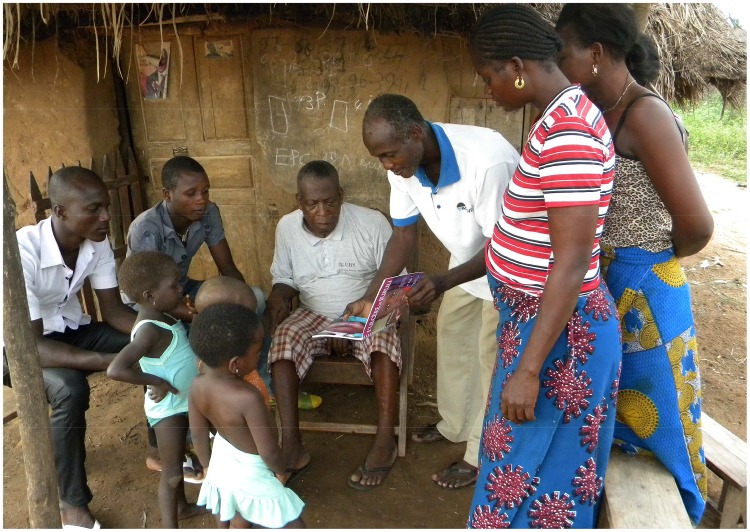
Community health volunteer in action.

Apart from the community health volunteers, teachers, family members, and former patients also proved to be crucial in the referral system. The methods used by the community health volunteers for case detection are active case finding and education. (Figure 3). In data compiled from all centers, community health volunteers were more successful in getting patients to the health center in an early stage of disease than other referral sources. However, when health center specific data was analyzed, this effect could not be seen anymore. This suggests that the quality and/or training of community health volunteers are not uniform throughout the area. Barriers in the early presentation by community health volunteers (e.g. logistics) could also be different throughout the area.

Visible functional limitations at the start of treatment were lower in patients referred by community health volunteers and patients referred by these volunteers less frequently visited a traditional healer than were patients in other referral groups. Earlier studies have shown that the therapeutic itinerary of patients goes through traditional healers before finally reporting to BU treatment centers [7], [15], [30]–[34]. In Benin, visiting a traditional healer is very common and this contributes to the patient delay in obtaining adequate treatment [7], [32]. These findings made us hypothesize that patients referred by the community health volunteers without a visit to the traditional healer may have saved money and also are likely to present to obtain treatment from the health center earlier. From the current study it cannot be concluded how to prioritize limited means to support referral. It is not known what would be more cost effective, e.g. to increase the number of community health volunteers or to expand training activities to enforce early case detection. At least, the results show that in a health system with limited human resources, continuous efforts to support the community health volunteers are important. In Bénin, different Non-governmental organizations (*NGOs*) support the PNLLUB to organize the training to the community health volunteers. Earlier study highlighted the role of the NGOs in such training [18]. The BU program organizes at least three times per year training for the volunteers, mainly focusing on the disease symptoms at the earlier stage. Some incentives such as t-shirts are given to the community health volunteers as motivation. They also get transport costs for case detection and are reimbursed for the transport. Sometimes, bikes or motorbikes are offered to improve mobility of the community health volunteers who report many patients. It is unknown whether compensation based on performance, e.g. per reported BU patient indeed leads to referral of more patients in an early stage of disease. The effect of such intervention of course varies per setting [35]. In some circumstances Community health volunteers may have a negative impact on the health system; in Benin some community health volunteers are known to have asked money from patients or to have made medical decisions they are not qualified to make. In the BU health system, this negative impact is limited because the community health workers are recruited from the best community health workers that were earlier involved in the control of dracunculiasis where they were also supervised regularly.

There is considerable heterogeneity between health systems. After adjustment for treatment center in the statistical model, no effect of the referral system on the early presentation was found. Especially in one health center a high percentage of patients (66.8%) reported at an advanced disease stage. A significant factor here may be that this is the only center without decentralization of care. Earlier studies demonstrate the importance of spatial access to health care in developing countries [36], [37]. Other explanations could be a difference in help seeking behavior of patients or a difference in the quality or frequency of community education activities.

Apart from the community health volunteers, the family member and the former patients played important roles in referring patients. Slightly more than 40% of patients were referred by these informally trained community members (22% by former patients and 18.7% by family members). This high number of referrals from family members and former patients may be an effect of the education patients receive during their stay in the hospital and the community education which is conducted as part of the national program. We therefore imagine that it could be useful to actively invite former patients and their family members to become community health workers by enforcing their role with continuing education and financial compensation of travel expenses.

We would not be inclined to prioritize the training to the traditional healers at this point in time. We tried earlier to expand the health promotion to some of the traditional healers. However, some of the trained traditional healers started performing BU excisions at home with inadvertent effects such as hemorrhage. As a result we abandoned this program.

In Benin, patients are treated on the basis of clinical diagnostic suspicion, according to WHO recommendations [9]. Furthermore, an effort is made to confirm cases by molecular methods using PCR. Indeed, about 70% of patients started on treatment had PCR-confirmed BU. Center C has the highest number of PCR-confirmed patients and had more power to detect statistically significant differences between referral systems. The PCR positivity rate among patients referred by the community health volunteers is not systematically lower than in patients referred by other systems. This suggests that for the medical doctor the clinical case mix presented by the community health volunteer is similar to the patients presented by the other referral systems.

The odds ratios used in the logistic regression are used to be able to adjust for treatment center but cannot be translated to relative risk ratios due to the high frequency of the outcome (late presentation). Other limitations are the lack of adequate assessment of functional limitation at the end of treatment and limitations due to retrospective design of the study. The Buruli Ulcer Functional Limitation Scale (BUFLS) [38], [39] could be included in the national program to easily assess this outcome measurement.

To conclude, the study findings indicate that community health volunteers are an important link in the control of BU in Benin. Future studies should address the most effective way to improve early case detection by interventions such as training, increase in the numbers of volunteers or development of a system with financial compensation based on performance in early case detection.

## Supporting Information

Checklist S1 **STROBE checklist.**
(DOC)
